# Supplementary material for: Pain, Complex Chronic Conditions and Potential Inappropriate Medication in People with Dementia. Lessons Learnt for Pain Treatment Plans Utilizing Data from the Veteran Health Administration
Source: Brain Sci. 2021 Jan 11;11(1):86. doi: 10.3390/brainsci11010086 (PMC7827274; doi:10.3390/brainsci11010086)
Supplement: Supplementary file 1 [file brainsci-11-00086-s001.pdf]

**Supplementary Table 1. ICD-9 Codes for Major Clinical Diagnoses Used in The Study**

| Domain | Diagnostic group* | ICD-9 Codes | NOTE |
|---|---|---|---|
| Primary exposure | Alzheimer's diseases and Related Dementia (ADRD) | 290 [290.0-290.3; 290.4-290.43; 290.8-290.9]<br>331 [331.0, 331.11, 331.19, 331.6, 331.7, 331.82, 331.89] | |
| Mental Health Comorbidities | Alcohol Use Disorder | 291 [291.1-291.5, 291.8 (291.81, 291.89), 291.9];<br>303 [303.01-303.03, 303.9 (303.91-303.93)];<br>305 [305.01-305.03];790.3;980 [980.8,980.9]; E860.0,E860.1,E860.8,E860.9 | |
| | Drug Use Disorder | 292 [292.1 (292.11, 292.12), 292.2];<br>304 [304.01-304.03, 304.1 (304.11-304.13), 304.2 (304.21-304.23), 304.3 (304.31-304.33), 304.4 (304.41-304.43), 304.5 (304.51-304.53), 304.6 (304.61-304.63), 304.7 (304.71-304.73), 304.8 (304.81-304.83), 304.9 (304.91-304.93)];<br>305.2 (305.21-305.23), 305.3 (305.31-305.33), 305.4 (305.41-305.43), 305.5 (305.51-305.53), 305.6 (305.61-305.63), 305.7 (305.71-305.73), 305.8 (305.81-305.83), 305.9 (305.91-305.93), 305.1X | |
| | Major Depressive Disorders | 296.2 (296.21-296.26), 296.3 (296.31-296.36) | |
| | Post Traumatic Stress Disorder | 309.81 | |
| | Anxiety Disorders | 300 [300.01, 300.02, 300.09];799.2 | |

| | | | |
|---|---|---|---|
| | Bipolar Disorders | 296 [296.01-296.06, 296.1 (296.11-296.16), 296.4 (296.41-296.46), 296.5 (296.51-296.56), 296.6 (296.61-296.66), 296.7 (296.71-296.76), 296.8 (296.81, 296.89), 296.9, 296.99]; V11.1 | Combined with a few other rare conditions into *"Other psychiatric diagnoses"* |
| Medical comorbidities | Delirium | 293.0 (Delirium/Transient Mental Disorders); 780.09 (Unspecified Delirium). | |
| | Coronary Artery Disease | 410 [410.01, 410.02, 410.1 (410.11, 410.12), 410.2 (410.21, 410.22), 410.3 (410.31, 410.32), 410.4 (410.41, 410.42), 410.5 (410.51, 410.52), 410.6 (410.61, 410.62), 410.7 (410.71, 410.72), 410.8 (410.81, 410.82), 410.9 (410.91, 410.92)]; 411 [411.1, 411.8 (411.81, 411.89)]; 412; 413 [413.1, 413.9];414 [414.01, 414.8, 414.9];V45.81 | |
| | Congestive Heart Failure | 402.01,402.11,402.91; 404.01,404.03,404.11,404.13,404.91,404.93; 428 [428.1,428.9,428.2x,428.3x,428.4x] | |
| | Chronic Obstructive Pulmonary Disorder and Allied Conditions | 490.0<br>491.0-491.9<br>492.0-492.8<br>493.0-493.92<br>494.0-494.1<br>496. | |

| | | |
|---|---|---|
| Diabetes | 250 [250.01-250.03,250.1 (250.11-250.13), 250.2 (250.21-250.23), 250.3 (250.31-250.33), 250.4 (250.41-250.43), 250.5 (250.51-250.53),250.6 (250.61-250.63), 250.7 (250.71-250.73),250.8 (250.81-250.83), 250.9 (250.91-250.93)];357.2 | |
| Hypertension | 401 [401.1, 401.9];<br>402 [402.01, 402.1, 402.11, 402.9, 402.91];<br>404.1, 404.9;<br>405 [405.01, 405.09, 405.1 (405.11, 405.19), 405.9, 405.91, 405.99];<br>437.2; 403.X; 404.x;405.x | |
| Chronic Liver disease | 570.0<br>571.0-571.9<br>572.0-572.8<br>573.0-573.9 | |
| Chronic Renal failure | 581.0-581.9<br>582.0-582.9<br>583.0-583.9<br>585.0-585.9<br>586.0<br>587.0<br>588.0-588.9 | |
| Stroke or Cerebrovascular Disease | 433.0 – 433.91<br>434.0- 434.91<br>435.1-435.9<br>436.0<br>437.0-437.9 | |

| | | | |
|---|---|---|---|
| | | 438.0-438.9 | |
| | Parkinson's disease | 332.0<br>332.1 | |
